# Gender Equality and the Global Gender Gap in Life Expectancy: An Exploratory Analysis of 152 Countries

**DOI:** 10.34172/ijhpm.2020.192

**Published:** 2020-10-14

**Authors:** José Tomás Mateos, José Fernández-Sáez, Jorge Marcos-Marcos, Carlos Álvarez-Dardet, Clare Bambra, Jennie Popay, Kedar Baral, Connie Musolino, Fran Baum

**Affiliations:** ^1^Department of Nursing and Physiotherapy, University of Lleida, Lleida, Spain.; ^2^Public Health Research Group, University of Alicante, Alicante, Spain.; ^3^Unitat de Suport a la Recerca Terres de l´Ebre, Fundació Institut Universitari per a la recerca a l’Atenció Primària de Salut Jordi Gol i Gurina (IDIAPJGol), Tortosa, Spain.; ^4^Unidat de Recerca, Gerència Territorial Terres de l´Ebre, Institut Catalá de la Salut, Tortosa, Spain.; ^5^Facultat de Enfermería, Campus Terres de l´Ebre, Universitat Rovira i Virgili, Tortosa, Spain.; ^6^Department of Health Psychology, University of Alicante, Alicante, Spain.; ^7^University Research Institute for Gender Studies, University of Alicante, Alicante, Spain.; ^8^Biomedical Research Networking Center for Epidemiology and Public Health (CIBERESP), Madrid, Spain.; ^9^Institute of Health & Society, Newcastle University, Newcastle upon Tyne, UK.; ^10^Division of Health Research, Lancaster University, Lancaster, UK.; ^11^Department of Community Health Sciences, Patan Academy of Health Sciences, Kathmandu, Nepal.; ^12^Southgate Institute for Health, Society & Equity, Flinders University, Adelaide, SA, Australia.

**Keywords:** Life Expectancy, Gender Gap, Gender Equality

## Abstract

When looking at life expectancy (LE) by sex, women live longer than men in all countries. Biological factors alone do not explain gender differences in LE, and examining structural differences may help illuminate other explanatory factors. The aim of this research is to analyse the influence of gender inequality on the gender gap in LE globally. We have carried out a regression analysis between the gender gap in relativised LE and the UN Gender Inequality Index (GII), with a sensitivity analysis conducted for its three dimensions, stratified by the six World Health Organization (WHO) regions. We adjusted the model by taking into consideration gross national income (GNI), democratic status and rural population. The results indicated a positive association for the European region (ß=0.184) and the Americas (ß=0.136) in our adjusted model. Conversely, for the African region, the relations between gender equality and the LE gender gap were found to be negative (ß=-0.125). The findings suggest that in the WHO European region and the Americas, greater gender equality leads to a narrowing of the gender LE gap, while it has a contrary relationship in Africa. We suggest that this could be because only higher scores in the GII between men and women show health benefits.

## Background

 Life expectancy (LE) at birth is an indicator traditionally used to assess overall socio-economic development, particularly the health status of populations, and serves as a comparator between states and regions. Contrary to many other health indicators, LE at birth shows an advantage for women in all countries and regions.^1^ Globally, between 1950 and 2017, LE for women increased from 52.9 to 75.6 years, while for men it increased from 48.1 to 70.5 years.^2^ This advantage in LE for women is recent – only since the middle of the 20th century have women surpassed men.^3^

 Traditionally, there have been attempts to explain this trend in two ways; a biological and a non-biological explanation. From a biological perspective it has been argued that women are biologically superior, possibly due to genetic or hormonal differences.^4,5^ That is to say, in equal socio-economic and healthcare conditions, women should – on biological grounds – have a higher LE than men.^6^ Although this line of research has contributed to a greater understanding of the gap between women and men, it provides a very limited explanation for the cross-sectional variations. Conversely, the non-biological approach is focused on social and behavioural factors. For example, the lengthening of women’s LE has been attributed to the reduction of maternal mortality due to medical innovation and women’s increased access to healthcare. However, this has been experienced differently across regions, in particular in many lower-income countries where women still do not have access to basic maternal healthcare.^7,8^ The gender gap in LE could also be ascribed to social differences, such as men’s greater propensity to risk-taking behaviours and harmful lifestyles.^9^ Men are also less likely to access healthcare services and are more likely to present late with symptoms.^10,11^ Additionally, as a consequence of often being the main breadwinner, they may also be more exposed to the ill-health effects of work, workplace hierarchies or unemployment.^12,13^ More recently, mainly in high-income countries, the gender gap in LE has decreased, partly because of the rapid increase in men’s LE and the slowdown in the increase of women’s LE.^14,15^

 These inequalities in health-related exposures and outcomes can be explained by gender relations, including the social construction of masculinity, which can contribute to men’s poor health outcomes and higher premature mortality.^13,16^ However, the relationship between gender and health is especially complex due to the different ways in which it can manifest itself, being a key element in the establishment of power relations.^17^ Due to these power dynamics, men globally, in all income settings, are often less at risk of experiencing poverty than women and have more ‘control’ over their lives.^18^

 Gender inequalities are embodied within social structures including political and economic institutions and health and social policies.^19,20^ In this paper we work within the boundaries of global data sets which present gendered data in terms of the biological categories of male and female, and thus we acknowledge that this can mask the diverse experiences of sex and gender identity. It is not our intention to reinforce gender binaries and heteronormativity,^21^ but to examine how these constructions contribute to gender inequalities and gendered experiences of health. Examining the health gap from a gender equality perspective allows a nuanced analysis of the differences and similarities in the behaviours and experiences of men and women, which may influence health outcomes. In this sense, the scientific literature has shown that the health of both men and women is not only different, but can also be inequitable, and the result of modifiable social factors such as social policies or distribution of resources and is therefore unfair.^22-24^ For example, the increasing participation of women in paid work and public life has led, for many reasons, to better health outcomes for women themselves and their children, including greater access and control over economic resources.^25-27^

 Policies that promote greater gender equality, therefore, have the potential to modify the gender gap in LE.^22,23^ Indeed, previous research shows positive correlations between gender equality and LE in both women and men, both in Europe^28^ and other regions.^29^ Meanwhile, some scholars highlight that if increased gender equality results only in women increasingly entering spheres traditionally occupied by men, such as the labour force and public representation, there could be diminishing gains, or losses, in health: equalising the health advantages and disadvantages of employment, but also increasing risky behaviours in women.^16,30^ However, if men then begin adopting the roles traditionally undertaken by women (eg, responsibility for child care), population health benefits are likely to rise again; provided that it is supported through increased social policy provision.^22,23^ Thus, it is plausible to argue that in countries with greater gender equality, the gender LE gap may be narrower than in those with less equality.

 However, knowledge is very limited about how gender equality impacts on this association in the wider context of high, middle and low-income countries.^31^ Even if the analysis of the gender gap in LE is not in itself an indicator of population health, it may facilitate insights on gender inequalities in health and possible opportunities for improvement. Increasing scientific knowledge of this relationship could help to determine how structural gender inequities contribute to the LE gap between men and women globally. We hypothesised that greater gender equality entails a narrowing in the gender gap in LE. Thus, the analysis reported here aimed to examine the association of gender equality and the gender gap in LE worldwide using the Gender Inequality Index (GII). It was undertaken by members of the Punching Above their Weight (PAW) Research Network, which aims to determine the structural factors that drive differences in LE. Specifically, PAW focuses on political determinants and social inequalities, including gender.^32^

## Methods

 We created a database using 2017 information from 152 countries with available gender disaggregated LE data, as well as data for gender equality, gross national income (GNI), democratic status and rural population. We extracted the data described below from reports published by different official and international organisations.

###  Variables Description

 Our dependent variable was the relative gender gap in LE with respect to men’s LE. We did this to avoid the masking caused by the absolute difference of female to male LE. That is to say, we considered that to compare countries with similar absolute differences in LE by sex, but very different general LE, could produce misleading results. This effect was accounted for if we relativised the gender gap to men’s LE using the following equation: ((Women’s LE – Men’s LE)/Men’s LE). This relative variable correlates 95.1% with the absolute difference variable, which shows a good representation of the gender gap in LE. The data on LE were extracted from the Human Development Index.^18^

 Our main independent variable was gender equality, represented through the GII used by the United Nations Development Programme.^33^ The GII rank goes from 0 to 1: 0 being the most equitable situation between women and men and 1 the most unequal situation. This is a composite index comprising three dimensions: (*a*) Reproductive Health (maternal mortality ratio; adolescent birth rate); (*b*) Empowerment (female and male population with at least secondary education; female and male shares of parliamentary seats); and (*c*) Labour Market Participation (female and male participation rates). Each dimension was measured positively, that is, an increase means an improvement in each.^33^

 We also included three further variables that could be expected to have an impact on gender equality and LE, and therefore could be confounding. Firstly, we introduced the GNI per capita, extracted from the World Bank database.^34^ This variable was introduced in the logarithmic form to simplify the understanding of results. Secondly, we introduced the democratic status of each country, extracted from the *Freedom in the World* annual report.^35^ These provide composite indices to compare civil liberties and political rights, with each country scoring from 0 to 4 depending on the presence or absence of 25 different items. We introduced this dimension into the analysis as previous research has shown that the influence democracy has on several health outcomes could also be a determining factor in the relationship between gender equality and LE.^36,37^ Thirdly, we introduced the percentage of the rural population of each country from the World Bank estimation.^34^ This indicator refers to people living in rural areas and is calculated as the difference between total population and urban population. Inhabitants in rural areas have less access to healthcare services than people living in urban areas, especially in low-income countries.^38^ This is one reason why people living in rural and remote areas have lower LE and poorer health outcomes.^39^

###  Analytical Procedure

 Firstly, we described the gender differences in LE between countries and separately analysed the relationship of each independent variable with the relative gender gap in LE. We performed a descriptive analysis of the means and their confidence intervals (95% CI), standard deviation and the minimum, median and maximum of the total mortality rates of men and women by age groups. In order to test if these rates were distributed according to a normal distribution, we calculated the Kolmogorov-Smirnov normality contrast. To analyse the association of the independent variables with the relative gender gap in LE, we calculated the Spearman rank correlation coefficient. Finally, we performed a linear regression analysis with three models: model 0 or univariate; model 1 adjusted by the logarithm of GNI; and model 2 adjusted by the logarithm of the GNI, the index of democratic status and rurality. We also replicated this analysis by the three dimensions of the GII. We stratified the same analysis by the six World Health Organization (WHO) regions: (*a*) African (AFRO); (*b*) Pan-American (PAHO); (*c*) South-East Asian (SEARO); (*d*) European (EURO); (*e*) Eastern Mediterranean (EMRO); and (*f*) Western Pacific (WPRO). In order to obtain smooth correlations and avoid problems with data interpretation we removed the Seychelles, Syrian Arab Republic, Russian Federation, Belarus, and El Salvador from the analysis, because their LE data were outliers.

## Results

 Our sample contained cross-sectional data from 152 countries which had available data for all variables, grouped by WHO regions: 35 from the AFRO region; 17 from the EMRO region; 47 from the EURO region; 27 from the PAHO region; 9 from the SEARO region; and 17 from the WPRO region. Regarding the GNI: five (3.3%) were low-income countries; 28 (18.4%) lower-middle; 37 (24.3%) upper middle; and 82 (53.9%) were high-income countries. Regarding the distribution of mortality by ages, we observed higher mortality rates for men in all the age groups. Women’s advantage in LE was seen in all countries. The countries which presented a larger relative gender gap in LE were Kazakhstan (0.15), Lithuania (0.15), Ukraine (0.15), Latvia (0.14) and Moldova (0.13). Countries with a smaller relative gender gap in LE were Bhutan (0.01), Burkina Faso (0.02), Sierra Leone (0.02), Algeria (0.03) and Bahrain (0.03). The remainder of the descriptive information for each country is shown in Supplementary file 1.

 Although overall the Spearman correlation between the relative gender gap in LE and the GII was not significant (ρ = -0.089, *P*= .270), the stratified analysis by WHO region showed two significant correlations in different ways (Figure). For the AFRO region, there was a negative correlation (ρ = -0.538 *P* = .001) which means that increasing gender inequality (GII = 1) produces a narrowing in the LE gap. Meanwhile, the EURO region showed a positive correlation, which means that this increase in gender inequality produces a widening in the LE gap.

**Figure:**
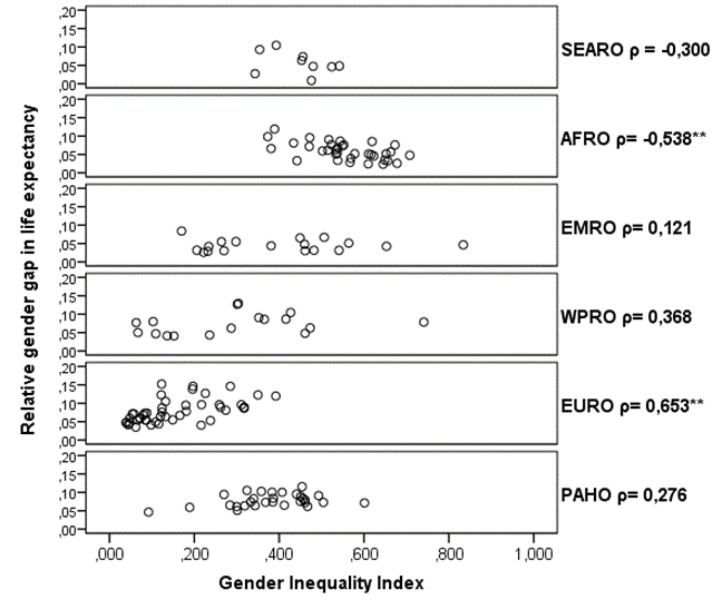
Spearman Correlation Between Relative Gender Gap in LE and GNI by WHO Region. Abbreviations: LE, life expectancy; GII, Gender Inequality Index; AFRO, African; PAHO, Pan-American; (c) SEARO, South-East Asian; EURO, European; EMRO, Eastern Mediterranean; WPRO, Western Pacific; WHO, World Health Organization.

**P value <.001.

 Regarding the separate analysis by the three GII dimensions, we obtained statistical significance for the Reproductive Health dimension in the AFRO, EURO and PAHO regions. These results were different depending on the region. In the AFRO region, there was a positive association between better reproductive health and a wider gender gap in LE (ρ = 0.350). By contrast, in the EURO (ρ = -0.630) and PAHO (ρ = -0.359) regions this association was negative, that is to say, an improvement in reproductive health entails a narrowing in the relative gender gap in LE.

 Attending to the regression model adjusted by GNI, democratic status and rurality, our hypothesis was held only for EURO and PAHO regions (Table 1). The negative correlation from the Spearman analysis persisted for AFRO and EMRO regions, but only the first one showed a statistical significance (*P* = .004). These significant relations remained in model 2, adding only GNI to the univariate model. The analysis without WHO region stratification showed a non-significant, negative relation between GII and the relative gender gap in LE (-0.040, *P* = .085). The democratic status of the countries did not show a significant relationship with the gender gap in LE.

**Table 1:** Regression Models With Gender Inequality Index by WHO Regions

**WHO Region**	**Model 0**			**Model 1**			**Model 2**		
	**ß**	***P***	**R** ^ 2 ^	**ß**	***P***	**R** ^ 2 ^	**ß**	***P***	**R** ^ 2 ^
AFRO	-0.153	**<.001**	32.3	-0.155	**.004**	28.0	-0.125	**.045**	23.9
EMRO	0.001	.980	0.1	-0.091	.082	14.6	-0.092	.126	1.1
EURO	0.185	**<.001**	32.7	0.139	**.046**	30.7	0.184	**.016**	33.5
PAHO	0.061	**.054**	13.1	0.129	**.011**	17.7	0.136	**.019**	11.7
SEARO	-0.152	.352	12.5	-0.136	.597	1.6	0.086	.861	3.5
WPRO	0.047	.245	8.9	-0.055	.402	17.5	-0.033	.649	23.8
Global	-0.017	.154	1.3	-0.047	**.036**	3.0	-0.040	.085	5.5

Abbreviations: AFRO, African; PAHO, Pan-American; (c) SEARO, South-East Asian; EURO, European; EMRO, Eastern Mediterranean; WPRO, Western Pacific; WHO, World Health Organization. Model 0: univariate. Model 1: adjusted by log(GNI). Model 2: adjusted by log(GNI), Freedom Status and Rurality (% of the total population).

 Attending to the separate analysis by the three GII dimensions, similarly to the Spearman correlation, there was an association between the improvement in the Reproductive Health dimension and the narrowing in the LE gender gap for the EURO and PAHO regions, but the association found in the AFRO region disappeared in the linear analysis (Table 2). Furthermore, we found a very weak relationship between the Empowerment dimension and the LE gap, such that increasing empowerment was associated with a widening in the LE gender gap for the global analysis (0.080, *P*<.001), but in the stratified analysis this relationship was present only for the EURO region (Table 2). The Labour Market dimension did not produce significant results in any case (Table 2).

**Table 2:** Regression Models With Each GII Dimension by WHO Regions

**WHO ** **Region**	**Reproductive Health Dimension**									**Labour Market Dimension**									**Empowerment Dimension**								
	**Model 0**			**Model 1**			**Model 2**			**Model 0**			**Model 1**			**Model 2**			**Model 0**			**Model 1**			**Model 2**		
	**ß**	***P***	**R** ^ 2 ^	**ß**	***P***	**R** ^ 2 ^	**ß**	***P***	**R** ^ 2 ^	**ß**	***P***	**R** ^ 2 ^	**ß**	***P***	**R** ^ 2 ^	**ß**	***P***	**R** ^ 2 ^	**ß**	***P***	**R** ^ 2 ^	**ß**	***P***	**R** ^ 2 ^	**ß**	***P***	**R** ^ 2 ^
AFRO	2.461	.060	8.2	1.600	.314	5.9	0.001	.999	4.2	0.007	1.000	0.1	0.053	.218	9.0	0.017	.731	13.1	0.036	.106	8.0	0.019	.473	6.1	0.054	.056	22.9
EMRO	-0.020	.893	0.1	0.344	.193	5.7	0.077	.427	0.7	-0.770	.068	20.6	-0.090	.126	10.0	-0.107	.139	-2.5	0.001	.967	0.0	0.029	.443	-2.4	0.030	.492	-19.0
EURO	**-0.579**	**<.001**	**38.3**	**-0.477**	**.006**	**36.5**	**-0.159**	**.007**	**35.8**	-0.027	.722	0.3	0.035	.599	24.8	0.047	.499	24.3	**0.147**	**.018**	**11.8**	**0.126**	**.019**	**33.3**	**0.119**	**.030**	**31.7**
PAHO	**-0.673**	**.014**	**19.0**	**-0.951**	**.002**	**25.6**	**-0.276**	**.012**	**15.1**	-0.048	.446	2.3	-0.049	.455	5.8	-0.054	.427	-10.8	-0.026	.032	4.3	-0.630	.100	3.4	-0.058	.173	-4.7
SEARO	0.535	.121	24.6	0.097	.961	18.1	-1.620	.279	13.1	-0.020	.860	0.5	0.006	.962	-24.9	-0.007	1.000	-6.4	0.085	.134	2.9	0.080	.196	7.6	0.104	.191	-1.0
WPRO	-0.124	.519	2.2	0.071	.803	3.4	0.029	.731	23.8	-0.066	.311	6.8	-0.072	.213	22.9	-0.077	.302	29.3	0.011	.784	0.5	**0.085**	**.037**	**37.4**	0.063	.152	35.1
Global	-0.055	.406	0.4	**-0.244**	**.012**	**3.0**	**-0.090**	**.005**	**8.6**	-0.015	.513	0.3	0.015	.544	0.3	-0.017	.244	6.4	**0.043**	**<.001**	**10.7**	**0.083**	**<.001**	**18.6**	**0.080**	**<.001**	**21.6**

Abbreviations: GII, Gender Inequality Index; AFRO, African; PAHO, Pan-American; (c) SEARO, South-East Asian; EURO, European; EMRO, Eastern Mediterranean; WPRO, Western Pacific; WHO, World Health Organization. Model 0: univariate, Model 1: adjusted by log(GNI), Model 2: adjusted by log(GNI), Freedom Status and Rurality (% of the total population).

## Discussion

 We performed a cross-sectional study of 152 countries to explore the influence of gender equality on the narrowing of the gender gap in LE. Our results showed some associations that open new possibilities for understanding women’s advantage in LE. Firstly, our data on mortality groupings by sex and age have showed that women’s survival advantage was present in all age groups. Furthermore, women’s LE advantage was common to all countries although the size of the gap varies considerably. Secondly, the different distributions among countries suggests that socioeconomic factors do influence the relative LE gap between men and women, confirming that it is not possible to attribute these differences only to biological factors. Thirdly, we identified different associations between gender equality and the gender gap in LE in different WHO regions.

 Our findings suggest that in the EURO and the PAHO regions, there is an association between greater gender equality and the narrowing of the relative LE gender gap, probably because of the increased LE of men. These results support Kolip and Lange’s study, which found a positive correlation between lower gender inequality and a smaller gender gap in LE in 28 European Union Member States.^28^ Conversely, we found a negative association in the AFRO region, with greater gender equality associated with a wider gap in LE between men and women. Similarly, Medalia and Chang found a heterogeneous association between gender equality and the gender gap in LE between low- and high-income countries.^29^ In their study, while in low-income countries an increase in gender equality led to women gaining more years of life than men, in high-income countries gender equality was associated with a narrowing in the gap. From the separate analysis between men and women, the authors found that the convergence between gender equality and LE in high-income countries could be due to gender equality benefiting men more than women, an argument developed below.^29^

 The above reasoning can be supported by the different stages of gender equality between countries and how this modulates the position and relations of men and women. Månsdotter and Deogan identified three main phases of gender equality progress from the perspective of public health which may help to unpack these regional differences.^40^ They argued that initial progress in gender equality leads to health gains through the improvement in females’ basic rights. If we attend to the mean in the GII by region analysed in our study, the AFRO region has the worst result of all (0.56 ± 0.01) while the EURO region (0.16 ± 0.01) and the PAHO region (0.38 ± 0.02) have the highest rates of equality. These findings could suggest that in those regions where higher stages of gender equality are reached, men increase their participation in domestic and caring responsibilities, and adopt better health behaviours and practices, which increases their LE. Alternatively, in regions with higher gender equality, women increase their participation in productive work and engage in risky health behaviours previously understood as ‘masculine,’ such as smoking or drinking alcohol, resulting in a slower increase in their LE.^25,30^ In the regions with less gender equality, changes in gender relations are still incipient, so women’s labour participation outside the private sphere is not as high and therefore it could be argued that their health is not as exposed to harmful risks as in countries with greater equality, which leads to a faster increase in their LE. On the other hand, men’s norms of masculinity may be closer to hegemonic ones, which leads to worse habits and greater exposure to risks, also slowing down the increase in their LE.

 Regarding the separate analysis of the dimensions of the GII, there seems to be a weak global association for both the dimensions of empowerment and reproductive health. The Reproductive Health dimension has a negative association with the relative gender gap in LE. We have to take into account that this dimension is the only one that does not include men’s situation, but reflects the differences between countries in women’s health status. Thus, this finding showed that an improvement in women’s health was associated with a reduction in the relative LE gender gap, mainly in high-income regions. Although they were not significant, the association in low-income regions was the opposite. This could be the reflection of a more extended improvement in health outcomes in high-income countries, that affects both women and men, and thus results in a greater LE for both and so less difference between men and women; for example, by decreasing neonatal and infant mortality rates amongst babies.^29^ In contrast, the dimension regarding Empowerment showed a positive association with the relative gender gap in LE in all regions except for the PAHO region. Even if the association was only significant for the EURO region, it remained significant at the global level. This dimension includes the educational level and political representation of both women and men. This result fits with previous literature that showed how greater gender equality in education led to lower women’s mortality.^29,41^

 Our results confirm that the relative gender LE differences were not fully explained by the variables used in our analysis and that investigation of other variables not included in our model is required. Firstly, the relationship between GNI and the relative gender gap in LE is not clear and should, therefore, be explored in greater depth, contrasting it with variables of economic and basic services equality. Furthermore, one conundrum of the gender gap that we have not explored is that LE is associated with poverty and women are more likely than men to live in poverty. Further, we have not taken into account income inequality as a factor potentially determining gender inequality in health (eg, the Gini coefficient). These potential relationships warrant further exploration.

 Some limitations must be taken into account when interpreting the results. Such a cross-sectional study is limited because it does not measure the past, whether the association was linear or overtime. LE is influenced by many different factors and we may not have captured the full complexity. It should be noted that our study takes an exploratory approach so we have included most of the societal factors that are known to affect this indicator. Although we have made an effort to remove the effects of confounding factors, we cannot rule out the effect of residual confounding. In addition, it should be noted that the indicator used to assess gender equality does not cover gendered norms and performance in the private domain. Although this may be a limitation of our study, the information available to compare countries in this sense is very scarce and so the GII is a good approximation for our purpose. Finally, GII includes maternal mortality, which could introduce a risk of conflation, as it affects women’s LE. The Reproductive health Dimension is composed of this ratio and adolescent birth rate, so we consider that this dimension is not based solely on the maternal mortality variable. Furthermore, we intend to explore the linear trend of relative LE with respect to GII, so if they are related it does not affect the meaning sought since collinearity would not occur.

## Conclusion

 This research is a preliminary attempt to understand the relationship between gender equality and the gender gap in LE globally. Our findings highlight the need to account for multiple dimensions of social structures and gender inequalities and how the impacts of gender equality on health may vary by regional or localised country conditions. These include the differential impact of ill-health processes in men and women, material infrastructures and services (including the distribution of income and wealth), how gender intersects with social functioning and other inequality axes such as race/ethnicity, social class, religion or sexuality.

## Acknowledgements

 We acknowledge the comments on this paper done by the attendants of the ‘Punching Above their Weight’ Network meeting in Cape Town and to Professor Andreu Nolasco for his suggestions on the analysis of data.

## Ethical issues

 Ethical approval is not required due to this study is based on secondary data.

## Competing interests

 Authors declare that they have no competing interests.

## Authors’ contributions

 JTM, JFS, JMM, and CAD led the conceptualisation of the study, design and interpretation of the results. CB, JP, CM, and FB assisted with the design. JTM and JFS provided the acquisition of the data and statistical analysis. JTM, JMM, and CAD drafting the manuscript. JFS, CB, JP, KB, CM, and FB critically reviewed the manuscript. All authors reviewed and approved the final manuscript.

## Funding

 This work was supported by the UK Academy of Medical Sciences Global Challenges Research Fund Networking Grant under Grant ACP Ref No. 102292 ‘Punching above their weight: Building capacity for research on some countries have better life expectancies than predicted by national income.’

## Authors’ affiliations

^1 ^Department of Nursing and Physiotherapy, University of Lleida, Lleida, Spain. ^2^Public Health Research Group, University of Alicante, Alicante, Spain. ^3^Unitat de Suport a la Recerca Terres de l´Ebre, Fundació Institut Universitari per a la recerca a l’Atenció Primària de Salut Jordi Gol i Gurina (IDIAPJGol), Tortosa, Spain. ^4^Unidat de Recerca, Gerència Territorial Terres de l´Ebre, Institut Catalá de la Salut, Tortosa, Spain. ^5^Facultat de Enfermería, Campus Terres de l´Ebre, Universitat Rovira i Virgili, Tortosa, Spain. ^6^Department of Health Psychology, University of Alicante, Alicante, Spain. ^7^University Research Institute for Gender Studies, University of Alicante, Alicante, Spain. ^8^Biomedical Research Networking Center for Epidemiology and Public Health (CIBERESP), Madrid, Spain. ^9^Institute of Health & Society, Newcastle University, Newcastle upon Tyne, UK. ^10^Division of Health Research, Lancaster University, Lancaster, UK. ^11^Department of Community Health Sciences, Patan Academy of Health Sciences, Kathmandu, Nepal. ^12^Southgate Institute for Health, Society & Equity, Flinders University, Adelaide, SA, Australia.

## Supplementary files


Supplementary file 1. List of included Countries, the WHO Region and the Information for Each Variable.
