# Supplementary material for: Gender Equality and the Global Gender Gap in Life Expectancy: An Exploratory Analysis of 152 Countries
Source: Int J Health Policy Manag. 2020 Oct 14;11(6):740–6. doi: 10.34172/ijhpm.2020.192 (PMC9309916; doi:10.34172/ijhpm.2020.192)
Supplement: Supplementary file 1 — List of included Countries, the WHO Region and the Information for Each Variable. [file ijhpm-11-740-s001.pdf]

Supplementary file 1. List of Included Countries, the WHO Region and the Information for Each Variable

Country	WHO Region	Gender Inequality Index	relative LE	GNI	log (GNI)	GNI Cat.	Freedom Status	Percent. of rural populat.
Afghanistan	EMRO	0,653	0,04	2000	3,3	Lower middle	5,5	74,8
Albania	EURO	0,238	0,05	12120	4,08	Upper middle	3	40,6
Algeria	AFRO	0,442	0,03	15050	4,18	High income	5,5	27,9
Argentina	PAHO	0,358	0,1	20270	4,31	High income	2	8,3
Armenia	EURO	0,262	0,09	10060	4	Upper middle	4,5	36,9
Australia	WPRO	0,109	0,05	47160	4,67	High income	1	14,1
Austria	EURO	0,071	0,06	52500	4,72	High income	1	41,9
Azerbaijan	EURO	0,318	0,09	16650	4,22	High income	6,5	44,7
Bahamas	PAHO	0,34	0,08	29790	4,47	High income	1	17,1
Bahrain	EMRO	0,222	0,03	42930	4,63	High income	6,5	10,8
Bangladesh	SEARO	0,542	0,05	4040	3,61	Upper middle	4	64,1
Barbados	PAHO	0,284	0,07	17830	4,25	High income	1	68,8
Belgium	EURO	0,048	0,06	48240	4,68	High income	1	2
Belize	PAHO	0,386	0,08	7890	3,9	Upper middle	1,5	54,4
Benin	AFRO	0,611	0,05	2260	3,35	Lower middle	2	53,2
Bhutan	SEARO	0,476	0,01	8850	3,95	Upper middle	3,5	59,8
Bolivia	PAHO	0,45	0,08	7330	3,87	Upper middle	3	30,9
Bosnia and Herzegovina	EURO	0,166	0,07	12880	4,11	High income	4	52,1
Botswana	AFRO	0,434	0,08	16990	4,23	High income	2,5	31,3
Brazil	PAHO	0,407	0,1	15160	4,18	High income	2	13,7
Brunei Darussalam	WPRO	0,236	0,04	83760	4,92	High income	5,5	22,7
Bulgaria	EURO	0,217	0,1	20500	4,31	High income	2	25,3
Burkina Faso	AFRO	0,61	0,02	1810	3,26	Lower middle	3,5	71,3
Burundi	AFRO	0,471	0,07	770	2,89	Low income	6,5	87,3
Cambodia	WPRO	0,473	0,06	3760	3,58	Lower middle	5,5	77
Cameroon	AFRO	0,569	0,04	3640	3,56	Lower middle	6	44,2
Canada	PAHO	0,092	0,05	46070	4,66	High income	1	18,7
C, African Republic	AFRO	0,673	0,08	730	2,86	Low income	7	59
Chad	AFRO	0,708	0,05	1920	3,28	Lower middle	6,5	77,1

Chile	PAHO	0,319	0,06	23670	4,37	High income	1	12,5
China	WPRO	0,152	0,04	16760	4,22	High income	6,5	42
Colombia	PAHO	0,383	0,1	14170	4,15	High income	3	19,6
Congo	AFRO	0,578	0,05	4880	3,69	Upper middle	6	33,5
Congo (DR)	AFRO	0,652	0,05	870	2,94	Low income	6,5	56,1
Costa Rica	PAHO	0,3	0,06	16100	4,21	High income	1	21,4
Côte d'Ivoire	AFRO	0,663	0,06	3820	3,58	Lower middle	4	49,7
Croatia	EURO	0,124	0,09	24700	4,39	High income	1,5	43,3
Cyprus	EURO	0,085	0,05	33610	4,53	High income	1	33,2
Czech Republic	EURO	0,124	0,08	34450	4,54	High income	1	26,3
Denmark	EURO	0,04	0,05	52390	4,72	High income	1	12,2
Dominican Republic	PAHO	0,451	0,09	15290	4,18	High income	3	19,7
Ecuador	PAHO	0,385	0,07	11350	4,05	Upper middle	3	36,3
Egypt	EMRO	0,449	0,07	11360	4,06	Upper middle	6	57,3
Estonia	EURO	0,122	0,12	31100	4,49	High income	1	31,3
Ethiopia	AFRO	0,502	0,06	1890	3,28	Lower middle	6,5	79,7
Fiji	WPRO	0,352	0,09	9090	3,96	Upper middle	3	44,3
Finland	EURO	0,058	0,07	45400	4,66	High income	1	14,7
France	EURO	0,083	0,07	43790	4,64	High income	1,5	19,8
Gabon	AFRO	0,534	0,05	17010	4,23	High income	6	11
Gambia	AFRO	0,623	0,05	1670	3,22	Lower middle	4,5	39,4
Georgia	EURO	0,35	0,12	10120	4,01	Upper middle	3	41,8
Germany	EURO	0,072	0,06	51680	4,71	High income	1	22,7
Ghana	AFRO	0,538	0,03	4490	3,65	Upper middle	1,5	44,6
Greece	EURO	0,12	0,06	27620	4,44	High income	2	21,3
Guatemala	PAHO	0,493	0,09	8000	3,9	Upper middle	4	49,3
Guyana	PAHO	0,504	0,07	8120	3,91	Upper middle	2,5	73,5
Haiti	PAHO	0,601	0,07	1830	3,26	Lower middle	5	45,7
Honduras	PAHO	0,461	0,07	4630	3,67	Upper middle	4	43,5
Hungary	EURO	0,259	0,1	26960	4,43	High income	2,5	28,9
Iceland	EURO	0,062	0,04	53280	4,73	High income	1	6,2
India	SEARO	0,524	0,05	7060	3,85	Upper middle	2,5	66,4
Indonesia	SEARO	0,453	0,06	11900	4,08	Upper middle	3	45,3
Iran	EMRO	0,461	0,03	21010	4,32	High income	6	25,6
Iraq	EMRO	0,506	0,07	17010	4,23	High income	5,5	29,7

Ireland	EURO	0,109	0,05	61910	4,79	High income	1	37,1
Israel	EURO	0,098	0,04	37910	4,58	High income	2	7,7
Italy	EURO	0,087	0,05	39640	4,6	High income	1	29,9
Jamaica	PAHO	0,412	0,06	8690	3,94	Upper middle	2,5	44,6
Japan	WPRO	0,103	0,08	44850	4,65	High income	1	8,5
Jordan	EMRO	0,46	0,05	9110	3,96	Upper middle	5	9,3
Kazakhstan	EURO	0,197	0,15	23440	4,37	High income	6	42,7
Kenya	AFRO	0,549	0,07	3250	3,51	Lower middle	4	73,4
Korea (Republic of)	WPRO	0,063	0,08	38340	4,58	High income	2	18,5
Kuwait	EMRO	0,27	0,03	83310	4,92	High income	5	0
Kyrgyzstan	EURO	0,392	0,12	3620	3,56	Lower middle	5	63,9
Lao PDR	WPRO	0,461	0,05	6650	3,82	Upper middle	6,5	65,6
Latvia	EURO	0,196	0,14	27400	4,44	High income	2	31,9
Lebanon	EMRO	0,381	0,04	14690	4,17	High income	5	11,6
Lesotho	AFRO	0,544	0,09	3510	3,55	Lower middle	3	72,3
Liberia	AFRO	0,656	0,03	710	2,85	Low income	3	49,3
Libya	EMRO	0,17	0,08	19940	4,3	High income	6,5	20,2
Lithuania	EURO	0,123	0,15	31030	4,49	High income	1	32,5
Luxembourg	EURO	0,066	0,05	72690	4,86	High income	1	9,3
Malawi	AFRO	0,619	0,09	1180	3,07	Lower middle	3	83,3
Macedonia	EURO	0,149	0,05	14590	4,16	High income	3,5	42,3
Malaysia	WPRO	0,287	0,06	28650	4,46	High income	4	24,6
Maldives	SEARO	0,343	0,03	15350	4,19	High income	5	60,6
Mali	AFRO	0,678	0,03	2160	3,33	Lower middle	4,5	58,4
Malta	EURO	0,216	0,04	36740	4,57	High income	1	5,5
Mauritania	AFRO	0,617	0,05	3900	3,59	Lower middle	5,5	47,2
Mauritius	AFRO	0,373	0,1	22570	4,35	High income	1,5	59,2
Mexico	PAHO	0,343	0,06	17840	4,25	High income	3	20,1
Moldova	EURO	0,226	0,13	6060	3,78	Upper middle	3	57,4
Mongolia	WPRO	0,301	0,13	11170	4,05	Upper middle	1,5	31,6
Montenegro	EURO	0,132	0,06	19150	4,28	High income	3	33,5
Morocco	EMRO	0,482	0,03	8060	3,91	Upper middle	5	38,1
Mozambique	AFRO	0,552	0,08	1200	3,08	Lower middle	4	64,5
Myanmar	SEARO	0,456	0,07	5830	3,77	Upper middle	5	69,7
Namibia	AFRO	0,472	0,1	10320	4,01	Upper middle	2	51

Nepal	SEARO	0,48	0,05	2710	3,43	Lower middle	3,5	80,7
Netherlands	EURO	0,044	0,04	52200	4,72	High income	1	8,9
New Zealand	WPRO	0,136	0,04	39740	4,6	High income	1	13,5
Nicaragua	PAHO	0,456	0,08	5680	3,75	Upper middle	4,5	41,7
Niger	AFRO	0,649	0,03	990	3	Low income	4	83,7
Norway	EURO	0,048	0,05	63980	4,81	High income	1	18,1
Oman	EMRO	0,264	0,05	40240	4,6	High income	5,5	16,4
Pakistan	EMRO	0,541	0,03	5830	3,77	Upper middle	4,5	63,6
Panama	PAHO	0,461	0,08	21890	4,34	High income	2	32,6
Papua New Guinea	WPRO	0,741	0,08	4040	3,61	Upper middle	3	86,9
Paraguay	PAHO	0,467	0,06	9180	3,96	Upper middle	3	38,7
Peru	PAHO	0,368	0,07	12890	4,11	High income	2,5	22,3
Philippines	WPRO	0,427	0,1	10030	4	Upper middle	3	53,3
Poland	EURO	0,132	0,1	27920	4,45	High income	1,5	39,9
Portugal	EURO	0,088	0,07	30980	4,49	High income	1	35,3
Qatar	EMRO	0,206	0,03	128060	5,11	High income	5,5	0,9
Romania	EURO	0,311	0,1	25150	4,4	High income	2	46,1
Rwanda	AFRO	0,381	0,07	1990	3,3	Lower middle	6	82,9
Saint Lucia	PAHO	0,333	0,07	13230	4,12	High income	1	81,4
Samoa	WPRO	0,365	0,09	6390	3,81	Upper middle	2	81,5
Sao Tome and Principe	AFRO	0,538	0,07	3370	3,53	Lower middle	2	28
Saudi Arabia	EMRO	0,234	0,04	54770	4,74	High income	7	16,4
Senegal	AFRO	0,515	0,06	2620	3,42	Lower middle	2	53,3
Serbia	EURO	0,181	0,08	14040	4,15	High income	2,5	44,1
Sierra Leone	AFRO	0,645	0,02	1480	3,17	Lower middle	3	58,4
Singapore	WPRO	0,067	0,05	90570	4,96	High income	4	0
Slovakia	EURO	0,18	0,09	30880	4,49	High income	1	46,2
Slovenia	EURO	0,054	0,07	33980	4,53	High income	1	45,7
South Africa	AFRO	0,389	0,12	13090	4,12	High income	2	34,2
Spain	EURO	0,08	0,07	37990	4,58	High income	1	19,9
Sri Lanka	SEARO	0,354	0,09	12470	4,1	High income	3,5	81,6
Sudan	EMRO	0,564	0,05	4480	3,65	Upper middle	7	65,6
Suriname	PAHO	0,441	0,1	14290	4,16	High income	2	34
Sweden	EURO	0,044	0,04	50980	4,71	High income	1	12,9
Switzerland	EURO	0,039	0,05	65610	4,82	High income	1	26,2

Tajikistan	EURO	0,317	0,09	3670	3,56	Lower middle	6,5	73
Tanzania	AFRO	0,537	0,05	2920	3,47	Lower middle	4	66,9
Thailand	SEARO	0,393	0,1	17090	4,23	High income	5,5	50,8
Togo	AFRO	0,567	0,03	1620	3,21	Lower middle	4	58,8
Tonga	WPRO	0,416	0,09	6050	3,78	Upper middle	2	76,8
Trinidad and Tobago	PAHO	0,324	0,11	30520	4,48	High income	2	46,8
Tunisia	EMRO	0,298	0,06	11490	4,06	Upper middle	2,5	31,4
Turkey	EURO	0,317	0,09	26160	4,42	High income	5,5	25,4
Uganda	AFRO	0,523	0,08	1820	3,26	Lower middle	5	76,8
Ukraine	EURO	0,285	0,15	8900	3,95	Upper middle	3	30,8
United Arab Emirates	EMRO	0,232	0,03	74410	4,87	High income	6,5	13,8
United Kingdom	EURO	0,116	0,04	42560	4,63	High income	1	16,9
United States	PAHO	0,189	0,06	60200	4,78	High income	1,5	17,9
Uruguay	PAHO	0,27	0,09	21870	4,34	High income	1	4,8
Uzbekistan	EURO	0,274	0,08	7130	3,85	Upper middle	7	49,5
Viet Nam	WPRO	0,304	0,13	6450	3,81	Upper middle	6	64,8
Zambia	AFRO	0,517	0,09	3920	3,59	Lower middle	4	57
Zimbabwe	AFRO	0,534	0,06	1850	3,27	Lower middle	5,5	67,8
